# Organic-based remediation of heavy metal-contaminated soils in the Taojia river basin affected by long-term non-ferrous mining and logging activities

**DOI:** 10.3389/fpls.2025.1486575

**Published:** 2025-03-18

**Authors:** Yan Zeng, Taimoor Hassan Farooq, Chenglin Yuan, Wang Li, Asma Farooq, Guangjun Wang, Yingchun Fang, Jun Wang, Wende Yan

**Affiliations:** ^1^ National Engineering Laboratory for Applied Technology in Forestry and Ecology in South China, Central South University of Forestry and Technology, Changsha, Hunan, China; ^2^ Bangor College, Central South University of Forestry and Technology, Changsha, Hunan, China; ^3^ Hunan Engineering and Technology Research Center of Heavy Pollution Industrial Wastewater Treatment and Recycling, Changsha, Hunan, China

**Keywords:** livestock manure, heavy metal, soil pollution, passivation remediation, ecological integrity, cleaner production

## Abstract

The upper reaches of the Taojia River have been impacted by unregulated logging linked to non-ferrous metal mining, resulting in significant mineral waste accumulation. Composting has shown promise in reducing heavy metal (HM) contamination in agricultural soils. This study included two segments: the first examined the effects of sheep manure (SM) and chicken manure (CM) with different concentrations on lead (Pb) dynamics in vegetable soils. The second applied the most effective method identified in segment one to assess Pb, cadmium (Cd), zinc (Zn), and copper (Cu) in soil, paddy, and straw in rice fields. Results showed that both compost types increased soil pH to mildly alkaline levels, with SM causing dose-dependent rises (insignificant between 2% and 5%) and CM inducing non-proportional alkalinity. CM compost significantly enhanced soil organic matter (SOM: 0.606–0.660 g/kg) compared to SM (0.414–0.495 g/kg). Total nitrogen (TN) spiked at 2% SM (0.172 g/kg) but plateaued until 10% SM (0.210 g/kg), while CM linearly increased TN with dosage. Total phosphorus (TP) rose proportionally with SM but remained unchanged under CM. For Pb immobilization, 5% SM reduced DTPA-Pb to 11.877 mg/kg, but 10% SM increased it (14.006 mg/kg), whereas 10% CM achieved optimal passivation (11.561 mg/kg). Correlation analyses linked compost dosage to SOM, TP, and available Pb (p < 0.05), with soil pH showing minimal direct influence. In rice fields, 10% CM elevated soil pH (7.10 vs. 6.71), TP, and total Zn/Cu/Pb/Cd but reduced Pb/Cd in paddy and straw. Heavy metal speciation revealed strong inter-state correlations (excluding exchangeable Pb), with soil pH and TP significantly influencing Zn, Cu, and Cd levels. These findings demonstrate CM compost’s dual role in improving fertility and mitigating Pb/Cd uptake, though Zn/Cu accumulation risks require careful management.

## 1 Introduction

With the rapid progress of urbanization, ecological and environmental challenges have grown increasingly severe, particularly concerning soil pollution caused by heavy metals (HMs) (Li C. et al., 2022; Alhammad et al., 2023). Human industrial activities, such as mining, metallurgy, and chemical industries, significantly contribute to the presence of HMs (Farooq et al., 2022; Xiang et al., 2022; Farooq et al., 2024), resulting in severe soil pollution in the surrounding areas. Soil microorganisms cannot biodegrade HMs and tend to accumulate in the soil (Xiang et al., 2022). The release of potentially toxic HMs from pyrite smelting processes can contaminate agricultural land, posing risks to human health (Zakaria et al., 2021). Furthermore, these pollutants can negatively impact soil enzyme activity and microorganism populations and disrupt the natural soil ecology (Gao et al., 2022), reducing plant diversity and influencing the evolution of soil microorganisms and associated functional genes (Alengebawy et al., 2021; Adnan et al., 2022). Additionally, HMs accumulated in the soil can be transferred to crops and plants and subsequently enter the food chain and living environments (Wang et al., 2022), potentially causing acute and chronic health issues in humans (Wang et al., 2021).

There are two primary strategies for addressing heavy HM pollution in soil. One involves directly immobilizing and modifying the chemical forms of HMs in the soil, aiming to reduce their mobility and bioavailability. This method often consists of the application of chemical agents or amendments such as lime, phosphates, or biochar to stabilize the metals and prevent them from being absorbed by plants or leaching into water sources (Wang et al., 2023). Altering the forms of HMs helps mitigate metal contamination’s immediate environmental and health risks. The other entails relocating and remediating the soil contaminated with HMs, known as *in-situ* and *ex-situ* remediation (Yang et al., 2021). Both strategies play a critical role in managing HM pollution, with the choice of approach often depending on site-specific conditions, including the type of metal pollutants and the extent of contamination. Various techniques are commonly employed for passivation restoration, including biological restoration (Rayne and Aula, 2020), physical restoration (Feng and Cheng, 2023), chemical restoration (Liu et al., 2018), and ecological restoration (Liu et al., 2018). Among these, chemical passivation restoration offers several advantages, such as a straightforward principle, short remediation time, and ease of operation (Hashimoto et al., 2008), making it an efficient solution for addressing heavy metal pollution in the soil environment (Liu et al., 2019; Luo et al., 2010; Shen et al., 2023; Wu et al., 2023).

As livestock and poultry breeding expands in scale, livestock and poultry manure production continues to rise, whereas untreated livestock leads to increasingly prominent ecological issues (Sabreena et al., 2022). Unplanned and untreated livestock manure usage has significantly contributed to soil pollution, with a relatively low resource utilization rate (Farooq et al., 2024). However, adequately treated and long-term application of livestock and poultry manure can improve soil physical properties, modify soil pH, and transform HMs and soil organic matter (SOM) (Silva-Castro et al., 2022), thereby remediating HM-polluted soil. The analysis of different animal manure composts revealed their potential for immobilizing HMs by examining their basic physical and chemical properties, elemental composition, and structural characteristics (Luo et al., 2022). When livestock and poultry manure compost is applied to Pb-polluted soil, it reduces the Pb (Lead) content by increasing the levels of humus and inorganic elements (Yang et al., 2019). Adding compost leads to a decrease in soil Pb content with increasing compost application rates. After compost addition, the content of Pb in the acid-extractable and reducible states decreases, while Pb shifts towards the oxidizable and stable residue states. This transformation occurs due to several mechanisms. Firstly, organic matter (OM) in the compost, particularly humic substances, can chelate and bind Pb, forming stable organic complexes that limit Pb mobility. Secondly, the increased pH and enhanced availability of phosphates and carbonates from the compost promote the precipitation of Pb as less soluble minerals, such as lead phosphates and carbonates. Additionally, microbial activity stimulated by the organic content of the compost may play a role in the biotransformation of Pb, facilitating its incorporation into more stable, less bioavailable forms (Zhang et al., 2019).

However, several important factors must be considered during the research process. Firstly, it should be noted that the feed additives used in livestock and poultry farming often contain HMs pollutants such as Pb, zinc (Zn), Copper (Cu), and cadmium (Cd). These HM elements can accumulate in livestock and poultry manure. Therefore, the compost derived from such manure needs to undergo appropriate treatment to ensure its safety before being used to remediate HM-contaminated soils (Mohammadpoor-Baltork et al., 2001; Li B. et al., 2022; Zheng et al., 2022). Secondly, it is essential to investigate the underlying mechanisms of animal manure composting, including its impact on changes in soil pH, cation exchange, adsorption, organic complexation, and precipitation with inorganic elements. Understanding these mechanisms will contribute to a comprehensive understanding of the composting process and its effects on soil remediation (Wang et al., 2019; Huang et al., 2022).

The Taojia River, originating from Xianghua Ridge and Thirty-six Bay, is a secondary tributary of the Xiangjiang River. This region is known as the primary concentration area for non-ferrous metal mines in Linwu, with a mining history spanning over 400 years. Due to the direct discharge of waste ballast and tailings from upstream mining activities, the Taojia River has experienced severe sedimentation, and the mining-related tailings and wastewater have caused significant pollution in the river. This study comprised two main parts: First, it examined the influence of sheep manure (SM) and chicken manure (CM) compost on the dynamics of Pb in vegetable fields within the Taojia River Basin. Second, it applied the most effective organic passivation method identified in the first part to study the morphology of Pb, Cd, Zn, and Cu in soil, paddy, and straw within rice fields. Given the HMs pollution in cultivated fields within the Taojia River Basin, this research explored the mechanism and effectiveness of utilizing livestock manure compost as a passivation agent to remediate heavy metal-contaminated soil.

This study hypothesized that both SM and CM manure composts would reduce Pb bioavailability in vegetable soils, with CM compost showing greater efficacy due to its generally higher nutrient content, which supports increased microbial activity and enhanced plant growth, and potentially higher OM content, which facilitates HM binding. Furthermore, it was hypothesized that an optimized compost treatment (identified through the vegetable soil experiments) would minimize HMs accumulation (Pb, Cd, Zn, Cu) in rice paddy and straw, while simultaneously improving soil quality and shifting HMs fractions towards less bioavailable forms. The findings from this study can provide a scientific and theoretical foundation for soil ecological restoration and the implementation of green planting practices in vegetable fields within the Taojia River Basin.

## 2 Materials and methods

### 2.1 Study area profile

**Figure 1:**
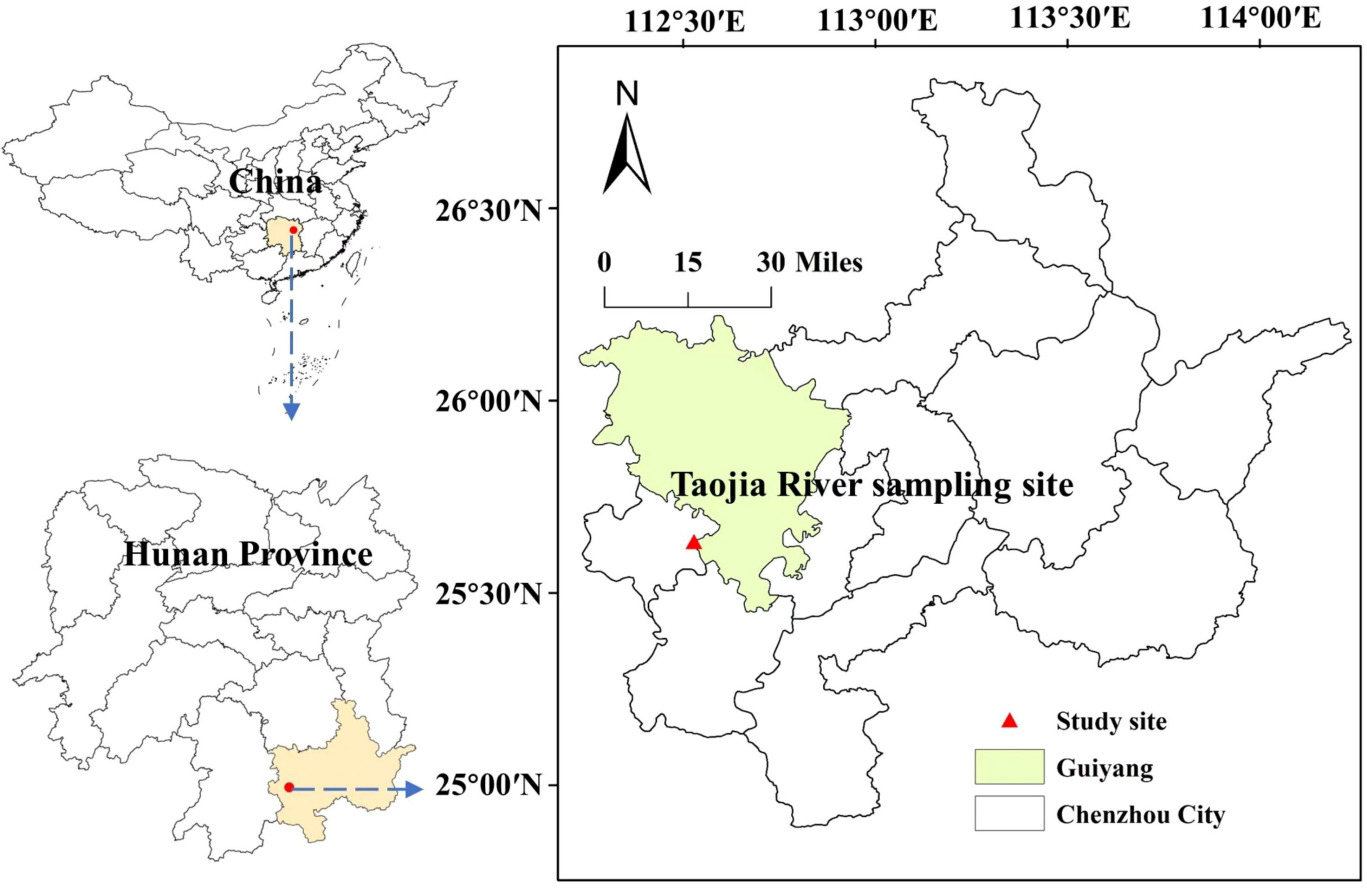
Location map of the study area (112°13 ‘26 “to 112°55’ 46” east longitude and 25°27 ‘15 “to 26°13’ 30” north latitude).

The research area is situated in the western region of Chenzhou City, Hunan provinvce, China, characterized by a sub-tropical humid monsoon climate. The geographic coordinates of the area range from 112°13’26” to 112°55’46” east longitude and 25°27’15” to 26°13’30” north latitude. The Taojia River, stretching approximately 17.59 kilometers, is a crucial water source for daily activities and production in the surrounding coastal areas. However, the upper reaches of the Taojia River have experienced sporadic and unregulated logging activities related to non-ferrous metal mining. As a result, a substantial amount of mineral waste stones and gravel have accumulated. During periods of flooding, these minerals are carried downstream, leading to the contamination of numerous cultivated soils situated along the river. Consequently, the ecological environment in the surrounding area has suffered significant damage (**Figure 1**).

### 2.2 Study segments

This study was divided into two segments. The first segment investigated how composting with SM and CM affected the dynamics of Pb in vegetable fields within the Taojia River Basin. In the second segment, we applied the most effective organic passivation method identified in the first part to examine the dynamics of Pb, Cd, Zn, and Cu in soil, paddy, and straw within rice fields. The selection of Pb, Zn, Cu, and Cd as HMs of environmental concern in the Taojia River Basin was based on our previous research findings (Yuan et al., 2022; He et al., 2023; Liu et al., 2024; Zeng et al., 2024). These metals were found to be of particular concern due to their higher concentrations in the affected soils, compared to other heavy metals. This made them a focus for assessing contamination and remediation efforts in the region.

This study employed a dual approach, utilizing both laboratory and field experiments. Vegetable soil, suitable for controlled laboratory settings, was chosen to investigate the mechanistic effects of compost amendments on HM immobilization under aboratory conditions. Complementing this, a field study was conducted using paddy soil, a prevalent soil type in the Taojia River Basin and an area susceptible to HM accumulation, which poses risks to agricultural productivity and food safety. This strategic selection aimed to bridge the gap between controlled experimental findings and real-world agricultural practices. By examining compost efficacy in both idealized laboratory conditions (vegetable soil) and a representative agricultural setting (paddy soil), the research sought a comprehensive understanding of its potential for HM remediation.

### 2.3 Experimental material and design for segment one

The SM and CM composts used in Segment One were sourced from the Shijiazhuang Biofertilizer Engineering and Technology Research Center products. The composting process involved three main stages: compost fermentation, manure storage tank fermentation, and biogas fermentation. We utilized fermented compost obtained from separately composting CM and SM with hay. During the fermentation process, the compost was regularly turned to ensure complete fermentation, and the temperature was maintained at 45°C for 15–30 days.

#### 2.3.1 Research design and methods for segment one

The experiment consisted of three groups: SM compost, CM compost, and a control group. Three different concentrations (2%, 5%, and 10%) were established within each group. The pH value, total nitrogen content (TN), total phosphorus content (TP), and OM of the two composts are presented in **Supplementary Table S1**. The experiment followed a sequence of steps. Initially, 500g of air-dried contaminated soil samples sourced from the vegetable field were meticulously weighed and placed into clean 1-liter plastic containers. Each experimental group consisted of 21 samples. Subsequently, the relevant compost for each experimental group was introduced to the soil samples, followed by thorough mixing.

To maintain the desired moisture level, water was carefully added to each sample until it reached and maintained a range of 60-70% of the field’s water-holding capacity. These prepared samples were then subjected to incubation at a constant temperature of 25 ± 2°C, and this condition was upheld for 15 days. The moisture levels were regularly monitored and adjusted throughout the incubation period by supplying additional water as needed, with adjustments made every other day. Each treatment was replicated three times. After the 15-day incubation period, multiple samples were combined and meticulously mixed to create a new composite sample for further analysis.

#### 2.3.2 Chemical analysis

The soil pH value, diethylenetriamine pentaacetic acid (DTPA)-extractable Pb (available Pb), total nitrogen content (TN), soil organic matter content (SOM), and total phosphorus content (TP) were analyzed using standardized methods. All analyses were performed in triplicate using and standard reference materials.

Soil pH was measured potentiometrically using the glass electrode method (Weaver and Wuensch, 2013). A 1:2.5 (w/v) soil-to-deionized water suspension was prepared, stirred for 30 minutes, and allowed to equilibrate for 1 hour before measurement using a calibrated pH meter. TN content was determined via the Kjeldahl nitrogen method (Thilakarathna and Raizada, 2018). Soil samples were digested with concentrated sulfuric acid (H_2_SO_4_) in the presence of a copper sulfate (CuSO_4_) catalyst. The liberated ammonia was distilled, trapped in boric acid (H_3_BO_3_), and quantified by titration with 0.01M HCl. SOM content was measured using the potassium dichromate (K_2_Cr_2_O_7_) oxidation method (Yuan et al., 2022). Briefly, 0.5g of soil was oxidized with 10mL of 0.5M K_2_Cr_2_O_7_ in concentrated H_2_SO_4_. The residual dichromate was titrated with 0.5M ferrous ammonium sulfate (FAS) using diphenylamine as an indicator. TP content was determined using the HClO_4_-H_2_SO_4_ digestion method (Saha et al., 2021). Soil samples (0.25g) were digested with a 3:1 mixture of H_2_SO_4_ and HClO_4_ at 350°C until a clear digestate was obtained. Phosphorus in the digestate was quantified colorimetrically at 880nm using the molybdenum blue method.

Available Pb was extracted using the DTPA method (Lindsay and Norvell, 1978). Air-dried soil (5.0g, ≤2mm) was mixed with 25mL of 0.005M DTPA extractant (pH 7.3) in a 100mL centrifuge tube. The suspension was shaken at 180 rpm for 2 hours on a reciprocating shaker, centrifuged at 4000×g for 10 minutes, and filtered through a 0.45μm membrane. Filtrate Pb concentrations were measured using flame atomic absorption spectrometry (FAAS, AA-3600F, Shanghai) at 283.3nm. Calibration standards (0–10 mg/L) and blanks were included, with recoveries of 92–105% for CRM GBW07401 (China National Standard Soil).

### 2.4 Experimental material and design for segment two

In the second phase of the study, two distinct treatments were implemented. In the first treatment, 10% CM compost, selected as the most effective passivator from the first segment, was incorporated into the soil—the other treatment served as a control, with no compost added. Two 20 x 20 square meter plots were prepared for this phase, and rice was cultivated in both and taken care of as per guidelines.

#### 2.4.1 Sample collection

Once the rice had reached maturity, removing stones, fallen leaves, and other debris from the soil surface was conducted. A portion of the soil sample was extracted using the point-centered quarter method. Sampling of the surface layer of soil (0-10 *cm*) took place at a total of 28 points, with the selection of 4-8 small square soil samples at each site, yielding an average of approximately 6-8 *kg* of soil collected per location. These soil samples were transported to the laboratory for subsequent analysis. Concurrently, rice and straw samples were also collected—these sample points corresponded to the soil sample points.

#### 2.4.2 Sample preparation

Before the chemical analysis, soil samples were air-dried and sieved through a 0.15 mm sieve. Rice and straw samples were oven-dried at 75°C for 48 h and processed by grinding them through a 100-mesh nylon sieve before being placed in plastic bags for preservation. These preserved soil samples were subsequently utilized to analyze soil pH, SOM, TN, TP, and HM morphology, namely Pb, zinc (Zn), cadmium (Cd), and copper (Cu). The resulting paddy and straw material were also used to analyze HMs morphology.

#### 2.4.3 Chemical analysis

The soil pH value, available state of Pb, TN, SOM, and TP contents of the soil samples were determined using specific methods mentioned in Segment One. Soil samples were placed on a hot plate, heated in a fume hood at low temperatures to micro-boiling (progressively heated to 140-160°C for about 60 min), and digested in aqua regia [HNO_3_: HCl (v/v) = 1:3] in preparation for analyzing HMs (Cu, Zn, Cd, and Pb) in the soil. For analyses of paddy and straw, digestion was performed with aqua regia [HNO_3_: HCl (v/v) = 1:4] on a hotplate at low temperature (80°C) for 30 min, then at high temperature (150°C) for 30 min, and finally again at high temperature (240°C-260°C, depending on the element to be determined).

### 2.5 Methods for heavy metal fractionation

The concentration of HM in the digested solution was determined by inductively coupled plasma mass spectrometry (ICP-MS) (HJ 803—2016). To assess the distribution of HMs among different chemical fractions, we employed a sequential extraction procedure based on the method established by Tessier et al. (1979). This method involves several steps: the exchangeable fraction was extracted using 1 M magnesium chloride (MgCl_2_), which targeted easily soluble metals; the carbonate-bound fraction was obtained with 1 M sodium acetate (NaOAc) at pH 5, allowing the identification of metals associated with carbonates; the iron and manganese oxides-bound fraction was extracted using 0.04 M hydrochloric acid (HCl), which released metals associated with these oxides; the organic matter-bound fraction was extracted using hydrogen peroxide (H_2_O_2_) followed by ammonium acetate (NH_4_OAc) at pH 4.5, which helps to determine metals bound to organic matter; and finally, the residual fraction was assessed by mineralizing the remaining residue with a mixture of nitric acid (HNO_3_) and hydrochloric acid (HCl). This comprehensive sequential extraction approach provides valuable insights into the mobility and bioavailability of heavy metals in the soil matrix (Tessier et al., 1979).

### 2.6 Statistical analysis

A one-way analysis of variance (ANOVA) was conducted at a 5% significance level to assess differences in descriptive statistics across compost treatments and their application rates (Hicks et al., 2022). Data were expressed as mean ± standard deviation (SD), and significant differences were evaluated using Tukey’s HSD *post hoc* test. Relationships between parameters were analyzed via Pearson correlation and visualized using hi-plots. Statistical analyses were performed with IBM SPSS Statistics (Version 21.0; IBM Corp., Armonk, NY, USA), while figures were generated using OriginPro software. In some cases, negative values for metal concentrations appeared in the results. These did not represent actual negative concentrations but instead reflected measurements below the detection limit of the analytical instruments, likely attributable to instrumental noise. Consequently, such values were interpreted as non-detectable concentrations (below the method’s detection threshold). While these values did not influence the overall trends or conclusions of the study, they were retained to ensure full transparency in data reporting—a standard practice in environmental research where trace metal levels may fall below reliable quantification limits.

## 3 Results

### 3.1 Soil chemical properties in vegetable-growing soils

The addition of SM compost increased soil pH, with the magnitude of the rise demonstrating a direct proportionality to the quantity applied. However, no significant difference in pH elevation was observed between the 2% and 5% SM treatments. CM compost also enhanced soil pH substantially, though the extent of improvement did not correlate proportionally with the application rate (**Table 1**). Overall, composted manure amendments resulted in a modest pH increase, maintaining the soil under slightly alkaline conditions.

**Table 1:** Concentration-dependent impacts of two manure composts on soil pH, organic matter, total nitrogen and total phosphorus content.

Rate	Sheep manure		Chicken manure		Control	
	Mean	SD	Mean	SD	Mean	SD
Soil pH						
2%	7.461 ^cB^	0.032	7.516 ^cA^	0.005	7.247 ^C^	0.063
5%	7.532 ^bB^	0.045	7.753 ^aA^	0.066	–	–
10%	7.651 ^aB^	0.036	7.703 ^bA^	0.045	–	–
Soil organic matter (g/kg)						
2%	0.495 ^aB^	0.006	0.660 ^aA^	0.012	0.297 ^C^	0.003
5%	0.420 ^bB^	0.002	0.606 ^aA^	0.011	–	–
10%	0.413 ^cB^	0.005	0.626 ^bA^	0.011	–	–
Soil total nitrogen (g/kg)						
2%	0.171 ^bB^	0.015	0.180 ^cA^	0.007	0.148 ^C^	0.001
5%	0.173 ^bB^	0.001	0.187 ^bA^	0.010	–	–
10%	0.210 ^aB^	0.009	0.215 ^aA^	0.009	–	–
Soil total phosphorus (g/kg)						
2%	1.407 ^cA^	0.166	1.325 ^cB^	0.109	1.175 ^C^	0.006
5%	1.738 ^bA^	0.082	1.358 ^bB^	0.109	–	–
10%	2.105 ^aA^	0.112	1.508 ^aB^	0.068	–	–

Lowercase letters denote significant differences among various concentrations of the same compost, while uppercase letters indicate significant differences between the same concentrations of different compost types.

SOM increased more markedly with CM compost than with SM compost. Additions of 2%, 5%, and 10% CM compost yielded SOM values of 0.660 g/kg, 0.606 g/kg, and 0.623 g/kg, respectively, whereas SM compost applications produced lower SOM levels of 0.495 g/kg, 0.421 g/kg, and 0.414 g/kg at equivalent rates (**Table 1**). TN content rose significantly to 0.172 g/kg in soil treated with 2% SM compost. However, increasing the SM compost rate to 5% did not induce further TN changes; a notable increase to 0.210 g/kg occurred only at the 10% application rate. In contrast, TN levels in CM compost-amended soils exhibited a dose-dependent proportionality (**Table 1**). TP content showed a substantial and linear increase in soils amended with SM compost, corresponding to the application rate. Conversely, CM compost additions did not significantly elevate soil TP content (**Table 1**).

### 3.2 DTPA-extractable Pb levels in vegetable-growing soils

**Figure 2:**
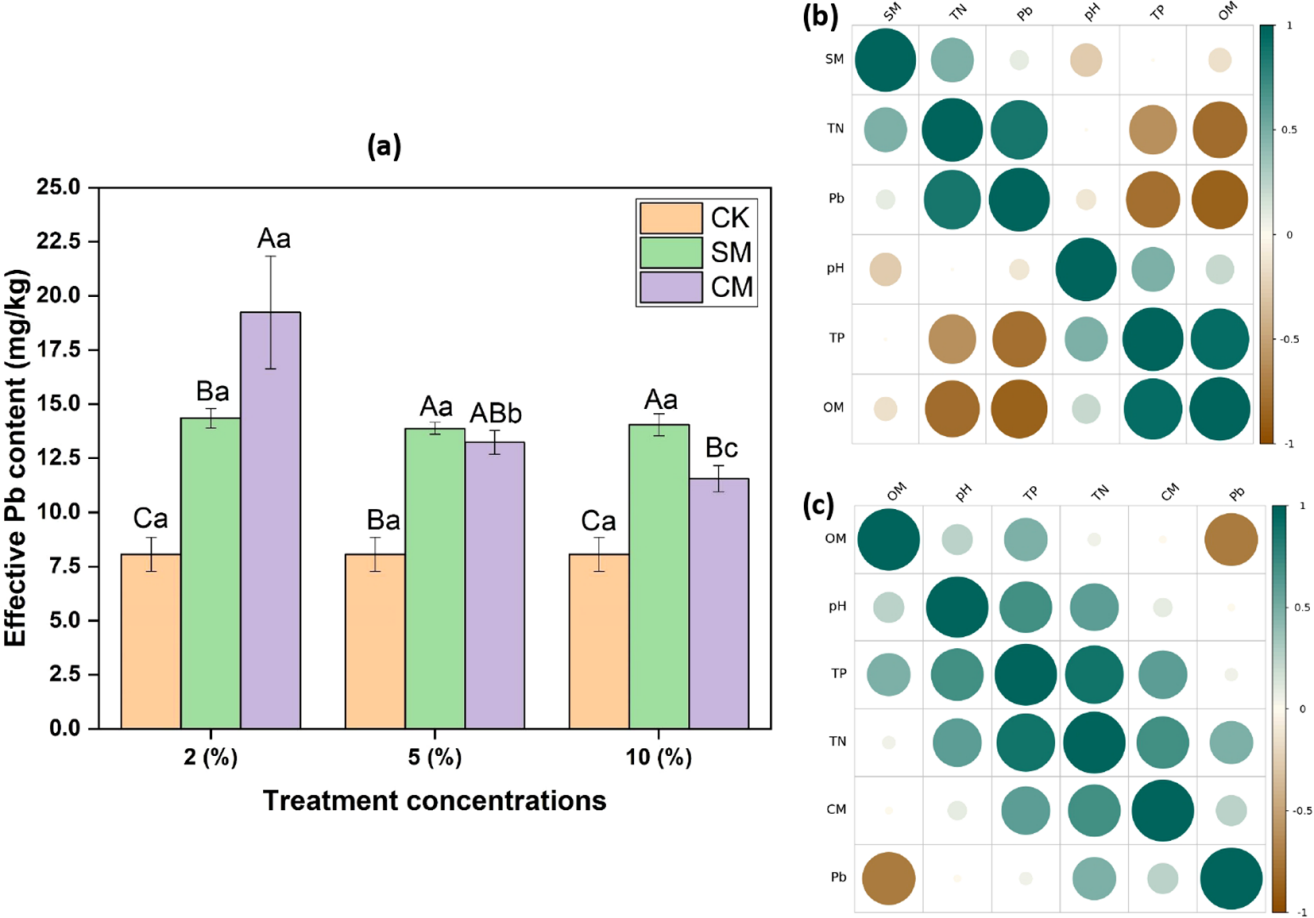
**(a)** Effects of two types of organic manure composts on soil DTPA-extractable lead (DTPA-Pb) content in a vegetable field. Different letters denote significant differences (p < 0.05). Lowercase letters indicate significant differences among different concentrations of the same compost, while uppercase letters indicate significant differences between the same concentrations of different compost types. **(b)** Pearson correlation between soil physical and chemical properties and DTPA-Pb content after the addition of sheep manure compost. **(c)** Pearson correlation between soil physical and chemical properties and DTPA-Pb content after the addition of chicken manure compost. In the correlation diagrams, darker colors and larger circles indicate stronger correlations; brown hues represent p-values closer to -1 (indicating a negative correlation), while green hues represent p-values closer to 1 (indicating a positive correlation).

At a 2% SM compost application rate, the soil’s DTPA-extractable Pb content reached 14.345 mg/kg. However, increasing the SM compost rate to 5% reduced the DTPA-extractable Pb content to 11.877 mg/kg. A further increase to 10% SM compost elevated the soil’s DTPA-extractable Pb content to 14.006 mg/kg. In contrast, the addition of 2% CM compost resulted in DTPA-extractable Pb content of 19.235 mg/kg. Beyond this rate, increasing the CM compost quantity produced a proportional decline in DTPA-extractable Pb levels. The highest passivation efficiency was achieved at a 10% CM compost rate, yielding an DTPA-extractable Pb content of 11.561 mg/kg (**Figure 2a**).

### 3.3 Relationship of soil properties with DTPA-extractable Pb in vegetable-growing soils

The relationship between soil pH levels and DTPA-extractable Pb demonstrated a weak correlation, suggesting that pH was not the primary driver of Pb activity. However, the introduction of SM compost revealed significant positive correlations between compost application and SOM (p < 0.05) and TP content (p < 0.05) (**Figure 2b**). Similarly, CM compost addition showed a significant positive correlation with SOM (p < 0.05). Additionally, a dose-dependent relationship emerged between the soil’s available Pb content and the compost application rate (p < 0.05) (**Figure 2c**).

### 3.4 Effects of 10% cattle manure compost on soil properties in rice fields

**Figure 3:**
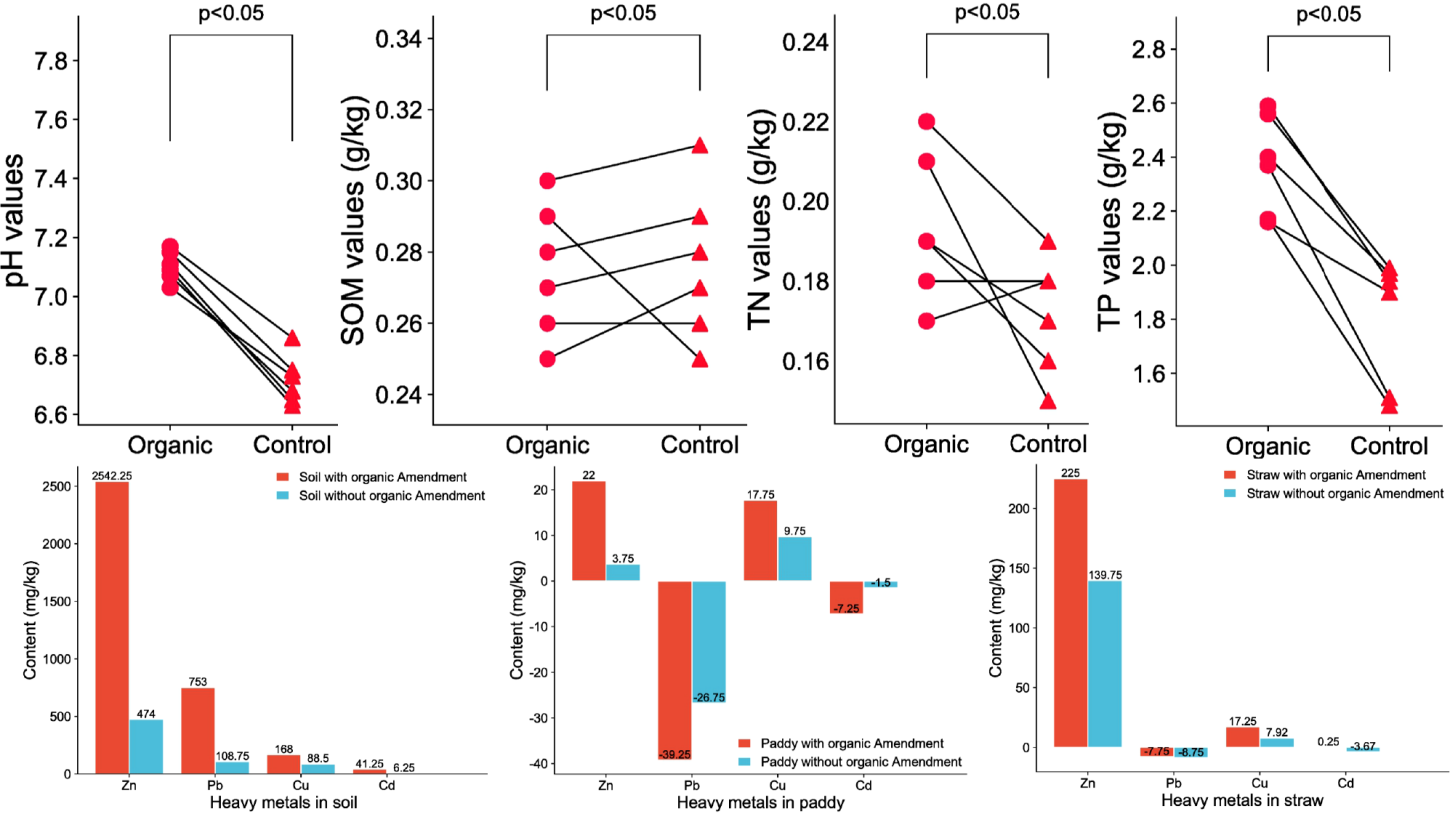
Comparison of soil physicochemical properties and heavy metal (Zn, Pb, Cu, and Cd) concentrations in soil, paddy, and straw under 10% chicken manure and control treatments in rice fields.

**Figure 3** demonstrated that the soil pH (7.10 ± 0.05) in the 10% CM compost treatment was significantly higher than that in the control (6.71 ± 0.07). No significant differences were observed in SOM between the two treatments. Soil TN content was marginally elevated under the 10% CM application, while soil TP content increased significantly in the 10% CM compost treatment compared to the control.

### 3.5 Effects of 10% cattle manure compost on heavy metal content in soil, paddy and straw

The comprehensive analysis of all HMs revealed that their levels were significantly elevated (p < 0.05) in soil treated with 10% CM compost compared to control group (**Figure 3**). In the paddy, a similar trend was observed for Zn and Cu, as their concentrations increased in parallel with their levels in the soil. In contrast, Pb and Cd concentrations in the paddy showed a more pronounced decrease under 10% CM compost treatment.

**Figure 4:**
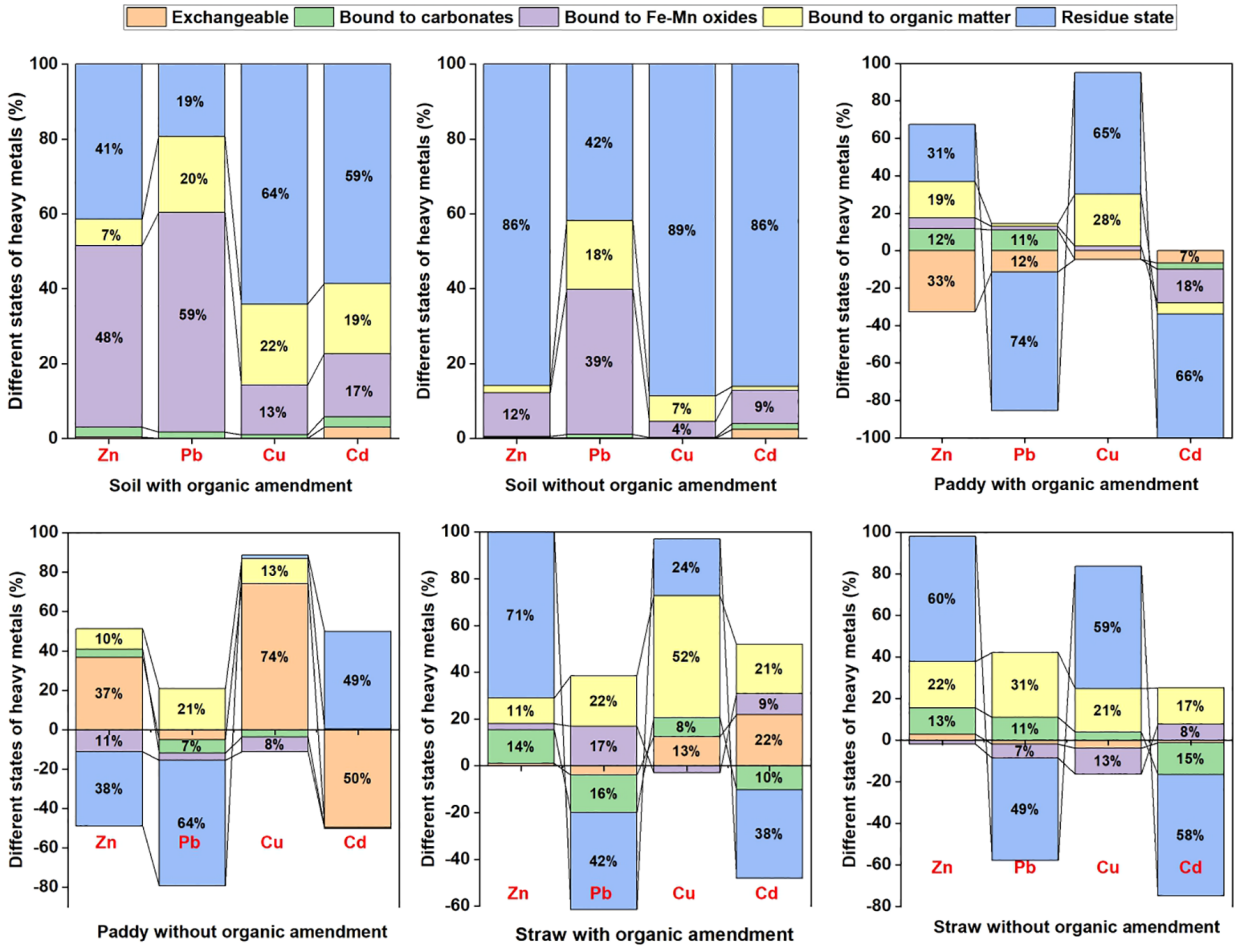
Percentage distribution and variations of heavy metal (Zn, Pb, Cu, and Cd) concentrations in soil, paddy, and straw under 10% chicken manure and control treatments in rice fields.

**Figure 5:**
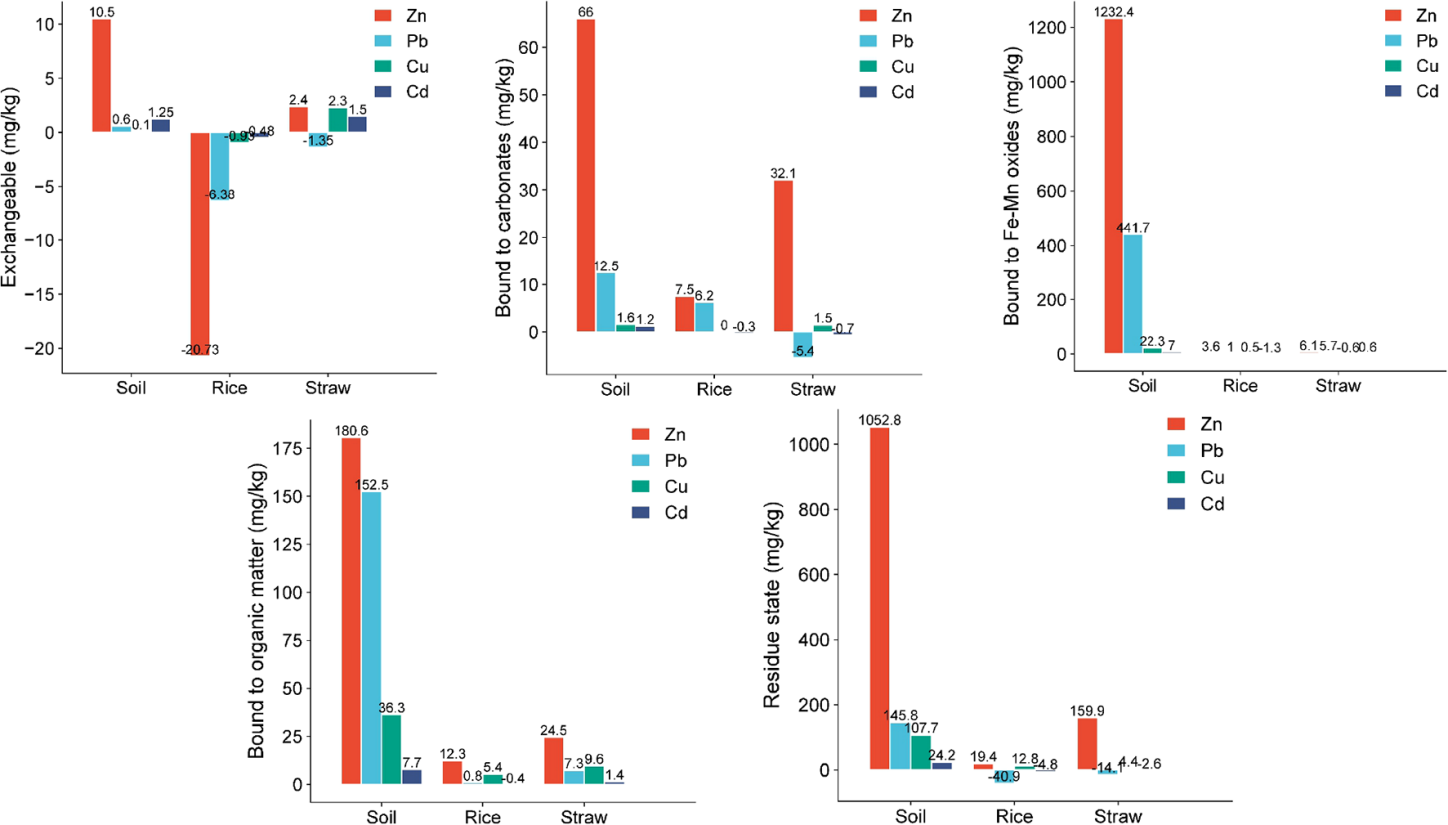
Concentrations of different states of heavy metals (Zn, Pb, Cu, and Cd) in soil, paddy, and straw after treatment with 10% chicken manure compost in rice fields.

**Figure 6:**
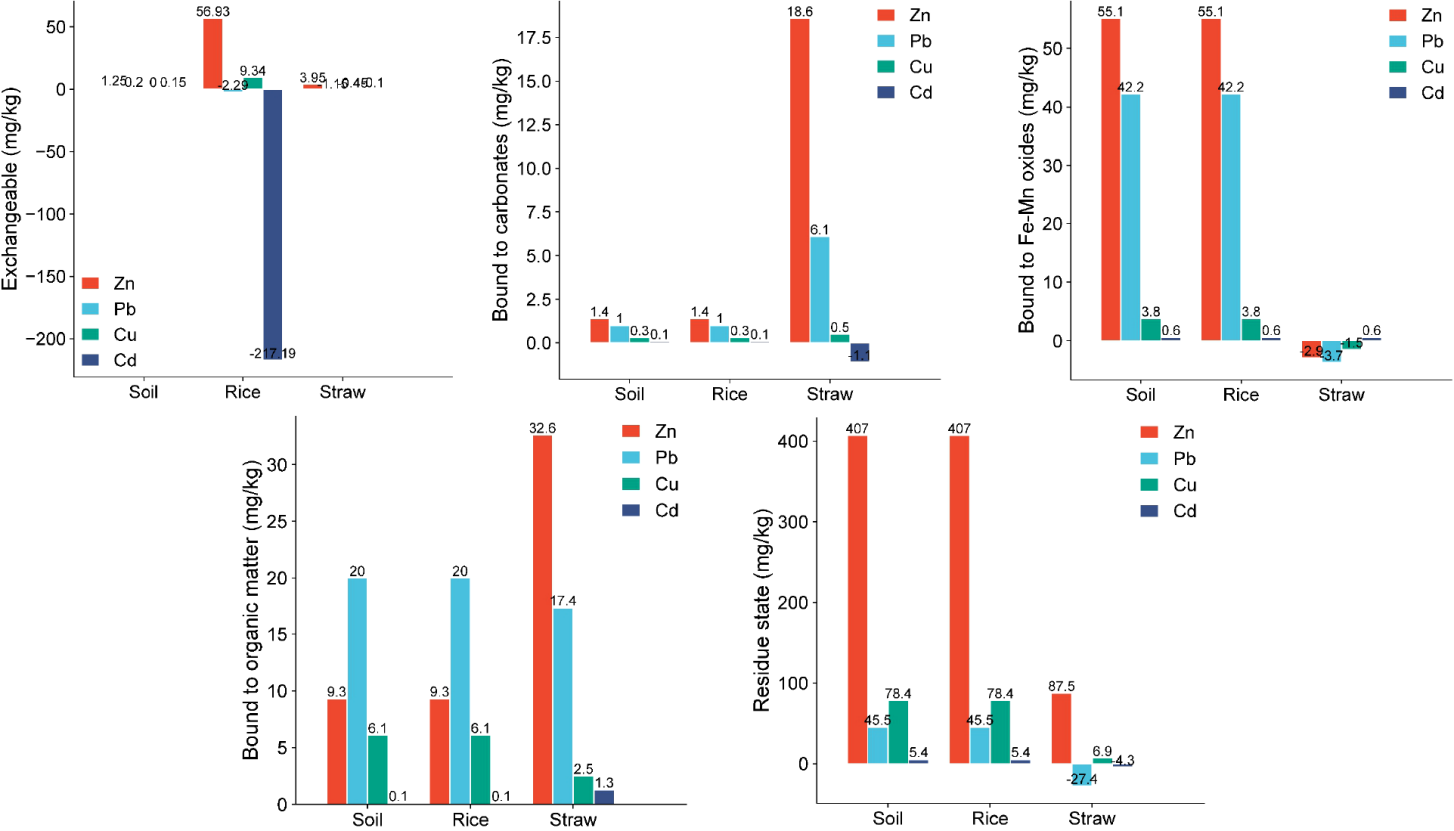
Concentrations of different states of heavy metals (Zn, Pb, Cu, and Cd) soil, paddy, and straw without any treatment (control) in rice fields.

In the straw, Zn and Cu concentrations were notably higher following 10% CM compost application, whereas Pb and Cd levels exhibited a significant decline compared to the control group. Regarding the different forms of HMs in soil, paddy, and straw, **Figure 4** presents their respective distribution percentages across various fractions, including the exchangeable, carbonate-bound, Fe-Mn oxides-bound, organic matter-bound, and residual states. The total contents of these HM fractions after 10% CM compost treatment and in untreated conditions are shown in **Figures 5** and **6**, respectively.

### 3.6 Relationship among different states of heavy metals in soil, paddy and straw

**Figure 7:**
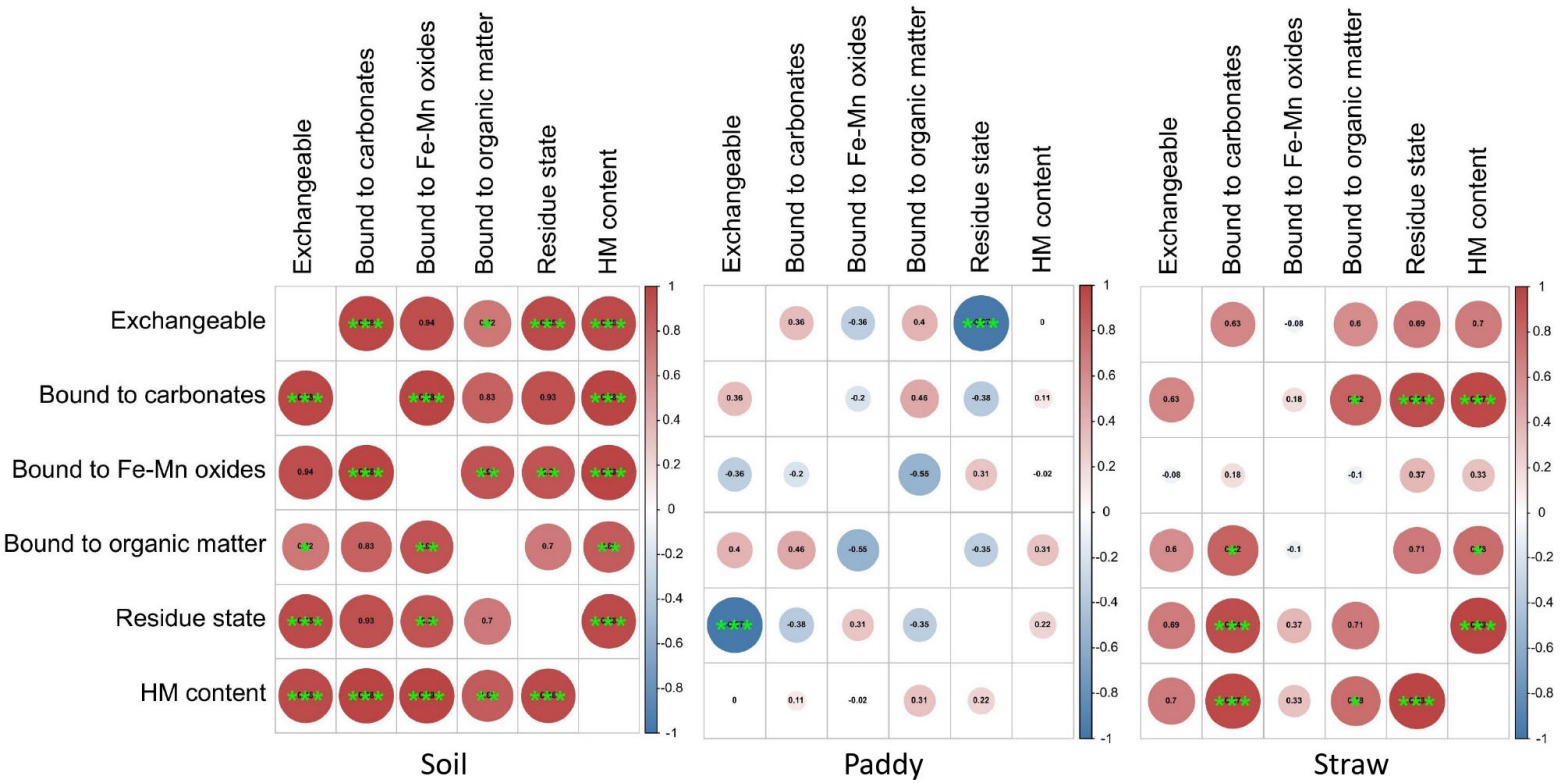
Correlation among different states of heavy metals across all the heavy metals in soil, paddy and straw. Significance levels are indicated by ,* , ** and *** for p < 0.05, p < 0.01, and p < 0.001, respectively.

**Figure 7a** showed the correlation among different states of HMs across different HMs and treatments. Except for the exchangeable state (p > 0.05), all other states of HMs exhibited statistically significant positive correlations with each other. Soil, paddy, and straw correlations were examined for a more in-depth analysis, as shown in **Figure 7a**. In soil, different states of HMs displayed positive correlations. However, in the case of straw, no clear correlation pattern was observed. Conversely, in paddy, a significant relationship was noted between various states of HMs, except for the exchangeable state and the residue state, which did not exhibit a significant correlation (**Figure 7b**).

### 3.7 Relationship between soil properties and heavy metals in soil, paddy and straw

**Figure 8:**
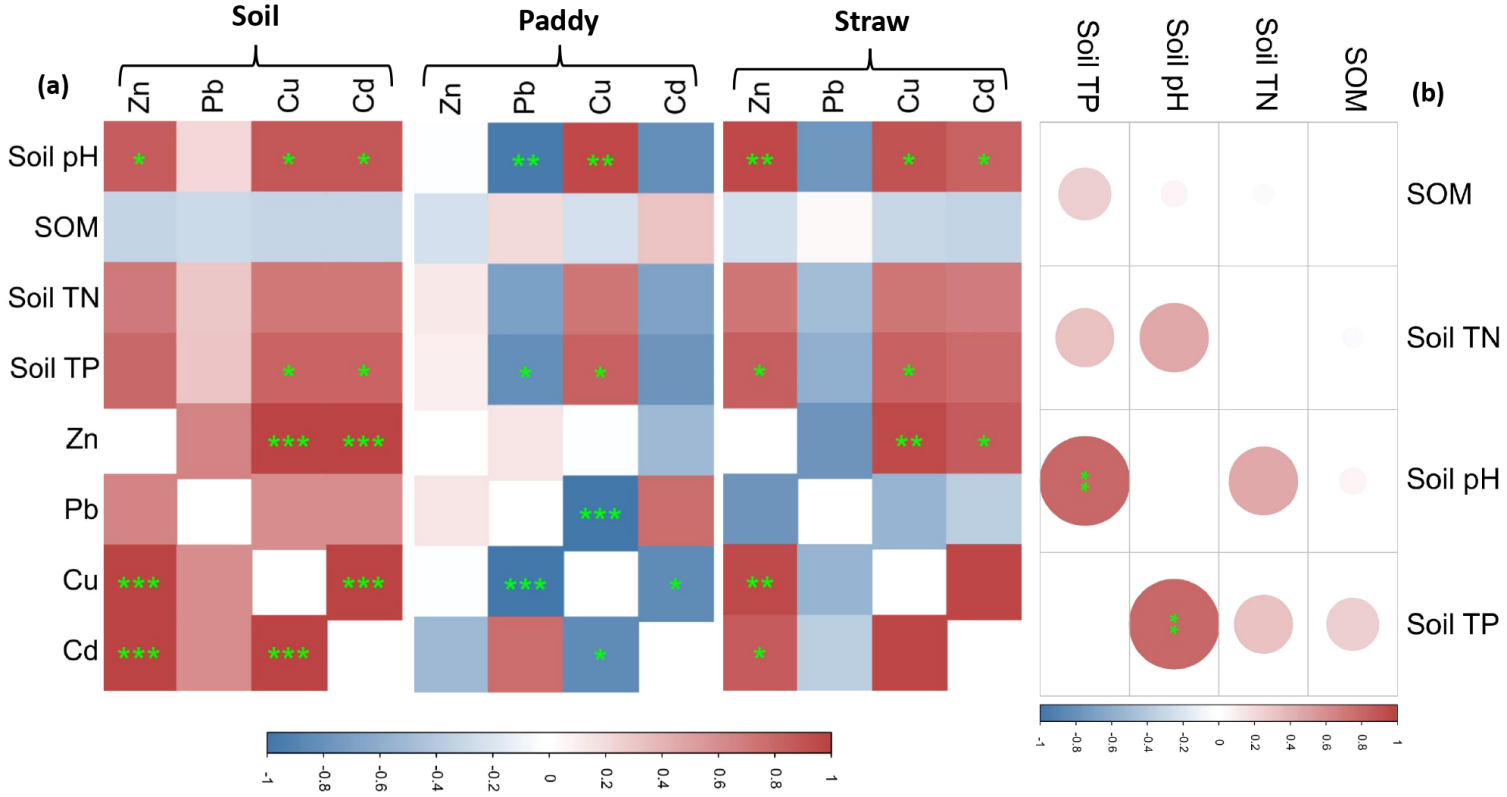
**(a)** Heavy metal interactions (Zn, Pb, Cu, Cd) in soil-paddy-straw systems and their linkages to soil physicochemical parameters, and **(b)** interdependence of soil properties (pH, SOM, TN, TP) in organic-amended and control rice fields. SOM, Soil Organic Matter; TN, Total Nitrogen; TP, Total Phosphorus. Significance levels are indicated by ,* , ** and *** for p < 0.05, p < 0.01, and p < 0.001, respectively.

In **Figure 8a**, we can observe the correlations among the total content of HMs in soil, paddy, and straw and their relationships with various soil properties for the 10% CM and CK treatments. Regarding soil properties, the soil pH showed significant correlations with Zn, Cu, and Cd content, while soil TP was associated with Cu and Cd. Notably, Zn exhibited a strong association with both Cu and Cd. Additionally, a robust relationship was found between Cu and Cd.

Concerning paddy, soil pH and TP were linked to Pb and Cu content, with Pb demonstrating a robust correlation with Cu. Likewise, there was a strong relationship between Cu and Cd. Regarding straw, soil pH and TP were associated with Zn and Cu content and soil pH was associated with Cd. Zn exhibited correlations with both Cu and Cd. It’s important to note that any relationships not mentioned were found to be statistically insignificant. **Figure 8b** presents the correlations among soil properties for the 10% CM and CK treatments. Except for the significant association between soil pH and soil TP, all other connections between soil properties were non-significant (p > 0.05).

## 4 Discussion

### 4.1 Effects of SM and CM composting on DTPA available-Pb in the vegetable soil

Remediating soil HM pollution through livestock manure compost involves both direct and indirect mechanisms (Xiao et al., 2020). Direct mechanisms include HM passivation through complexation, adsorption, precipitation, and redox reactions within the soil. Indirectly, CM compost alters key soil properties such as pH, OM, TN, and TP content. After a 15-day greenhouse culture, the available Pb content in the untreated vegateble soil sample was 8.062 mg/kg. However, upon adding the two types of compost, the available Pb content in the soil increased. During the greenhouse culture period, microbial activity within the soil samples played a multifaceted role in influencing HMs. Microbial processes such as enzymatic catalysis (Saha et al., 2021), adsorption, dissolution, and redox reactions (Bai et al., 2020; García-Carmona et al., 2017; Jacob et al., 2018) contributed to these changes. Additionally, microorganisms produced various organic acids, including formic acid, butyric acid, citric acid, malic acid, and lactic acid, which facilitated HM dissolution and complex formation with HM elements in the soil (Manikandan et al., 2023).

Moreover, microbial enzymes enhanced HM solubility by reducing metal oxides (Ayangbenro and Babalola, 2017). These mechanisms contributed to the observed increase in available Pb content following compost application (Silva et al., 2005; Gaur et al., 2021). However, when compost addition exceeded a critical threshold, the available Pb content began to decline. Our results indicated that adding 10% CM compost reduced available Pb by 58.09% compared to adding 2% CM compost. This reduction could be attributed to multiple mechanisms: an increase in OM enhanced cation-exchange capacity, promoting greater Pb ion binding. Additionally, organic compounds in compost formed stable Pb complexes, reducing solubility and mobility. Compost also stimulated microbial activity, leading to biosorption and Pb precipitation. Furthermore, changes in soil pH due to compost addition likely facilitated Pb precipitation into less soluble forms.

These findings suggest that adjusting the dosage of SM and CM compost, while considering soil physicochemical properties, can aid in mitigating and remediating Pb contamination. The application of organic manure compost to Pb-contaminated soil influenced HM dynamics through microbial processes, including enzymatic catalysis, which enhanced HM solubility. Consequently, fixed Pb in the soil was released, increasing available Pb content. In cases where small amounts of SM and CM compost were added, residual Pb partially dissolved from the soil. However, as compost addition increased, Pb transitioned from extractable and reducible states to a more stable residual state, leading to an increase in oxidizable and available Pb content. Livestock and poultry manure compost not only enriched the soil with humus and inorganic elements but also facilitated Pb complexation and precipitation, ultimately reducing its bioavailable concentration.

The addition of SM and CM compost significantly increased SOM content compared to the control, with the extent of increase correlating with compost dosage. SOM plays a key role in enhancing organic adsorption and chelation intensity, promoting HM precipitation and reducing their bioavailability (He et al., 2022). This transformation resulted in more HM elements forming insoluble complexes (Liu et al., 2018). The SM and CM composts used in this study contained varying SOM compositions, combined with inorganic compounds capable of forming mineral precipitates and binding HMs. These interactions effectively immobilized HMs in the soil, further limiting their bioavailability. Moreover, both composts exhibited minimal effects on soil pH, and the correlation between pH and DTPA-Pb was weak. This suggests that under weak alkaline conditions, soil pH plays a limited role in controlling available Pb content and is not the primary influencing factor (Farrell and Jones, 2009).

### 4.2 Effect of 10% CM on the soil biochemical traits in the rice field

Soil pH serves as a crucial indicator of the soil’s acidity or alkalinity. Where as SOM is a significant parameter in assessing land fertility, as it plays a pivotal role in supplying essential nutrients required for plant growth and development (Taskin et al., 2021). The introduction of a 10% CM led to a 5.7% increase in soil pH compared to the control group, demonstrating the effectiveness of this amendment. Interestingly, the addition of 10% CM compost did not result in any substantial difference in SOM compared to the control group.

TN and TP are vital nutrients for plant growth, and assessing their total quantities in the soil provided valuable insights into the soil’s nutrient supply capacity for fertilization (Francioli et al., 2016). The incorporation of CM compost significantly enhanced the N and P content in the soil. Compared to the control group, soil treated with 10% CM exhibited a notable 15.7% increase in TN and a substantial 30.6% boost in TP. This pattern aligned with the findings of Bożym and Siemiątkowski (2018), where the application of CM compost similarly resulted in a significant rise in soil pH. This effect was attributed to the alkaline nature of CM compost, which had a relatively high pH value and was applied in substantial quantities.

Notably, in China, the primary source of soil pollution in agricultural fields stemmed from inorganic fertilizers. While these fertilizers were essential for enhancing crop yields, they often led to the accumulation of harmful HMs and other toxic substances in the soil (Dong et al., 2021). Additionally, the unselective application of inorganic fertilizers altered soil pH and microbial activity, further exacerbating soil degradation (Saha et al., 2018). However, compost proved effective in mitigating HM contamination in soils, offering a sustainable alternative to the excessive use of inorganic fertilizers. A comparison of these results with those of other studies highlighted the gradual release of plant nutrients into the soil through the biodegradation of animal manure, suggesting that increasing the proportion of manure could enhance various soil quality attributes (Zhang et al., 2019; Iqbal et al., 2022).

### 4.3 Effect of 10% CM on heavy metals in rice field

High manure application rates consistently led to increased soil levels of HMs, namely Zn, Pb, Cu, and Cd. This trend aligned with the findings of previous studies (Kebrom et al., 2019). Following the addition of the 10% CM, the HM content, particularly Zn and Pb was notably higher in soil treated with CM than in the control group. The gap between Zn and Pb was particularly pronounced, indicating a significant accumulation of these HMs. Variations in the spatial distribution of HMs and dust deposition may have contributed to differences in soil HM content (Zhao et al., 2021).

HMs existed in various forms, each with distinct environmental behaviors and levels of toxicity. Among these forms, the exchangeable and carbonate-bound states exhibited high mobility and enhanced bioavailability, as highlighted by Zhang et al. (2018). Conversely, the iron-manganese oxide-bound state and the organic matter- and sulfide-bound state were relatively stable, with reduced bioavailability compared to the weak acid state, which represented a biologically potentially available form. When HMs were in the residue state, they demonstrated high stability and generally posed minimal risks to organisms and the environment, as their impact was usually insignificant.

**Figures 4**, **5**, and **6** illustrated the distribution of HM forms in the soil. Notably, Cu and Cd predominantly existed in the residue state, with Cu having the highest proportion, followed by Cd, Zn, and Pb. Conversely, Zn, Cu, and Cd primarily existed in the less favorable exchangeable form. Pb had the highest proportion among these four HMs in its exchangeable form. It was imperative to take timely measures to prevent the migration of Pb to crops, as doing so could help mitigate crop pollution (Jiang et al., 2022).

In this study, the addition of CM compost resulted in increased HM levels in the straw and paddy compared to the pre-compost application period. This increase may have been due to the nutrient-rich composition of CM compost, which enhanced overall plant growth and, consequently, facilitated greater uptake of HMs by plant tissues (Zhang et al., 2019). However, the exchangeable state of Zn, Pb, and Cu in the paddy decreased following compost application. This reduction was likely due to the binding of these HMs to organic matter in the compost, leading to the formation of stable complexes that rendered them less bioavailable.

Furthermore, the total contents of Pb and Cd in rice and straw declined, while Zn and Cu increased. The decreased levels suggested effective immobilization facilitated by the organic components of CM compost, which enhanced the retention of these HMs in less soluble forms (Saha et al., 2018). In contrast, the increased levels of Zn and Cu may have indicated improved nutrient availability due to compost application or a synergistic effect that enhanced the absorption of these HMs (Zhao et al., 2021). Correlation analysis revealed significant associations (p < 0.05) between paddy soil pH, TP, and Pb and Cd levels. These findings suggested that composting primarily reduced the accumulation of Cd and Pb in rice seeds and straw by elevating soil pH and TP, which aligned with (He et al., 2022).

Additionally, the application of CM compost notably increased the exchangeable Pb fraction in the soil but had a limited effect on Pb accumulation in rice grains. Therefore, CM compost could serve as a valuable soil amendment for enhancing soil fertility and reducing Cd and Pb accumulation in paddy fields (Tang et al., 2021). However, the potential risk of metal accumulation in the soil due to the repeated application of chicken manure compost should be carefully considered. Moreover, while straw return to the field is generally recognized as beneficial for soil enrichment (Glithero et al., 2013), it could also exacerbate the accumulation of HMs. In this rice-growing region, returning straw to the field should not be done uncritically. Given the enrichment of Zn and Cu in the straw, proper treatment of the straw was essential to prevent secondary pollution.

## 5 Conclusions

The application of SM and CM compost enhances soil fertility and mitigates HM contamination in arable soils, though their efficacy varies. CM compost demonstrated optimal Pb passivation at a 10% application rate, achieving the lowest DTPA-Pb content, while SM exhibited a practical threshold at 5% dosage, reducing Pb to 11.877 mg/kg before rebound at higher concentrations. CM compost outperformed SM in Pb immobilization, aligning with its superior enhancement of SOM and TP content, critical for long-term soil health. Both composts increased soil pH to mildly alkaline levels. In rice fields, 10% CM reduced Cd and Pb translocation to paddy and straw but elevated Zn and copper Cu concentrations, underscoring its dual role in risk mitigation and bioaccumulation potential. Correlation analyses confirmed compost dosage strongly influenced SOM accumulation, TP availability, and Pb immobilization, though soil pH had limited direct impact on HM activity. Despite these benefits, the long-term implications of Zn/Cu accumulation and HM speciation shifts (e.g., Fe-Mn oxide and organic-bound states) necessitate further study to ensure sustainable soil management. These insights provide actionable strategies for policymakers and farmers to balance soil remediation, crop safety, and ecological resilience in HM-contaminated agroecosystems. This approach not only reduces environmental risks but also enhances food safety, fostering interdisciplinary collaboration and supporting sustainable agricultural development. The present study did not include continuous manure compost application in either field or laboratory settings. Our research focused on assessing the immediate effects; however, we recognize that continuous application is crucial for understanding the long-term impacts and sustainability of manure compost use and should be investigated in future research.

## Data Availability

The original contributions presented in the study are included in the article/supplementary material. Further inquiries can be directed to the corresponding author.
