# Supplementary material for: Organic-based remediation of heavy metal-contaminated soils in the Taojia river basin affected by long-term non-ferrous mining and logging activities
Source: Front Plant Sci. 2025 Mar 18;16:1486575. doi: 10.3389/fpls.2025.1486575 (PMC11959093; doi:10.3389/fpls.2025.1486575)
Supplement: Supplementary file 1 [file Table1.docx]

**Supporting material**

**Table S1.** Physicochemical properties of compost used for the study of vegetable-grown soil. P_2_O_5_ is phosphorus pentoxide. Data are shown as the mean ± SD.

| Basic physical and chemical properties | Sheep manure | Chicken manure |
| --- | --- | --- |
| Moisture content θ (%) | 12.65 ± 0.20 | 18.29 ± 0.12 |
| pH | 7.40 ± 0.02 | 7.91 ± 0.02 |
| Organic matter (%) | 6.43 ± 0.30 | 8.85 ± 0.55 |
| Total nitrogen (g·kg^-1^) | 0.59 ± 0.32 | 0.66 ± 0.30 |
| Total Pb (mg·kg^-1^) | 9.58 ± 0.12 | 8.65 ± 0.05 |
| P_2_O_5_ (g·kg^-1^) | 22.20 ± 0.48 | 9.32 ± 1.08 |
